# Increased DHEAS and Decreased Total Testosterone Serum Levels in a Subset of Men with Early-Onset Androgenetic Alopecia: Does a Male PCOS-Equivalent Exist?

**DOI:** 10.1155/2020/1942126

**Published:** 2020-02-12

**Authors:** Rossella Cannarella, Rosita A. Condorelli, Federica Dall'Oglio, Sandro La Vignera, Laura M. Mongioì, Giuseppe Micali, Aldo E. Calogero

**Affiliations:** ^1^Department of Clinical and Experimental Medicine, 95123 Catania, Italy; ^2^Dermatology Clinic, University of Catania, 95123 Catania, Italy

## Abstract

**Background:**

Increased dehydroepiandrosterone sulfate (DHEAS) levels have been reported in men with early-onset (<35 years) androgenetic alopecia (AGA). It has been suggested that a male polycystic ovarian syndrome- (PCOS-) equivalent, defined as an endocrine syndrome with a metabolic background and a PCOS-like hormonal pattern, predisposing to type II diabetes mellitus (DM II), cardiovascular and prostate diseases later in life, may occur in at least a part of these men. The gonadal function, including sperm parameters and total testosterone (TT) levels, has been investigated in a low number of these men.

**Objective:**

The aim of the study was to assess gonadal and adrenal function in a subset of men with early-onset AGA and controls.

**Methods:**

43 men with early-onset AGA and 36 controls were screened for DHEAS, TT, glycaemia, insulin, gonadotropins, 17*α*-hydroxyprogesterone (17*α*-hydroxyprogesterone (17*n* = 21), as those with at least one of the following parameters: body mass index (BMI) >25 kg/m^2^, insulin resistance (IR), and/or SHBG <25 nmol/l.

**Results:**

Patients with early-onset AGA had higher mean (±SD) BMI (25.5 ± 3.8 vs. 23.7 ± 3.0 kg/m^2^; *P* < 0.05) and 17*α*-hydroxyprogesterone (17*P* < 0.05) and 17*P* < 0.05) and 17*P* < 0.05) and 17*P* < 0.05) and 17*μ*g/dl; *P* < 0.05) and 17*P* < 0.05) and 17*P* < 0.05) and 17*P* < 0.05) and 17

**Conclusion:**

Men with early-onset AGA and at least one among BMI >25 kg/m^2^, IR, and SHBG <25 nmol/l have increased DHEAS levels and a worse gonadal steroidogenesis. They might have a greater risk to develop gonadal dysfunction later in life. These criteria may be used to define male PCOS-equivalent.

## 1. Introduction

Male hypogonadism is defined as a gonadal dysfunction resulting in low total testosterone (TT) serum levels [1]. Dehydroepiandrosterone sulfate (DHEAS) is a molecule with a weak androgenic activity mainly secreted by the adrenal gland and, by a lesser extent, by the gonads, whose role in the pathogenesis of hypogonadism remains debated.

Meta-analytic data provide evidence for increased DHEAS levels and a worse glycolipid profile in male patients with early-onset (<35 years) androgenic alopecia (AGA), defined by a grade of alopecia higher than III according to the Hamilton–Norwood scale [2, 3], compared to controls [4]. Early-onset AGA may represent a feature of a male polycystic ovarian syndrome- (PCOS-) equivalent [5], a syndrome which causes gonadal dysfunction in the female gender.

The existence of a male PCOS-equivalent, whose features are summarized in Table 1, has been hypothesized based on the evidence of a genetic component in the etiology of female PCOS [6–10]. Accordingly, a higher prevalence of early-onset AGA has been observed in the first-degree male relatives of women with PCOS [11], in which hormonal, metabolic abnormalities [8, 12–14], and endothelial dysfunction [15] have been identified. Therefore, early-onset AGA has been investigated as a clinical sign of the male PCOS-equivalent [16–18]. In addition, a hormonal PCOS-like profile, consisting of decreased follicle-stimulating hormone (FSH), increased luteinizing hormone (LH), TT, androstenedione, and 17*α*-hydroxyprogesterone (17*α*OH-P) levels, hypertension, the presence of insulin resistance (IR), high body mass index (BMI), and low sex hormone binding globulin (SHBG), has been found in men with early-onset AGA [19–28].

A higher risk with aging to develop type II diabetes mellitus (T2DM) and coronary heart disease has also been observed in men with early-onset AGA [5]. Hence, AGA has been proposed as an independent predictor of mortality for T2DM and cardiovascular diseases (CVDs) [29]. These long-term issues resemble those known to be present in female PCOS [30–34]. They are also similar to those occurring in male hypogonadism [35, 36].

However, whether metabolic and cardiologic impairment is due to a concomitant hypogonadism in men with AGA is not known as studies assessing the gonadal function of these patients are lacking. In addition, likely female PCOS does not affect all women with hirsutism/acne and irregular menses, male PCOS-equivalent may occur only in a subset of men with early-onset AGA. Indeed, early-onset AGA has been estimated in approximately the 30% of men [37], whereas the prevalence of PCOS in women ranges between 4 and 7% [38–41]. As such, like in female PCOS, biochemical and/or clinical criteria are needed to identify a male PCOS-equivalent among men with early-onset AGA. Furthermore, the testicular function needs to be explored in these men.

Therefore, the aim of this study was to evaluate whether BMI >25 kg/m^2^, IR and/or SHBG <25 nmol/l could be used to identify the presence of a male PCOS-equivalent among men with early-onset AGA and to assess their gonadal function.

## 2. Methods

This case-control study was conducted at the Division of Andrology and Endocrinology and the Dermatology Clinic, of the teaching hospital “G. Rodolico,” University of Catania (Catania, Italy). The inclusion criteria were men with early-onset (before 35 years) AGA. The diagnosis of AGA was performed by clinical observation and by trichoscopy, a noninvasive *in vivo* technique that allows scalp and hair examination at high magnification (10–100x). In particular, trichoscopy was performed in the area delineated at the cross between the nose line and the ear implantation line, confirming the diagnosis of early-onset AGA by showing a hair diameter diversity >20%, a sign of progressive hair miniaturization [42].

Patients with at least one of the following were excluded: positive clinical history for cryptorchidism, testicular focal injury/injuries, testicular microlithiasis, varicocele, azoospermia, head injury, endocrine abnormalities (Cushing syndrome, acromegaly, and hypopituitarism), systemic diseases (kidney and/or liver diseases) and diabetes mellitus, consumption of alcohol and ingestion of drugs that may interfere with spermatogenesis (immunosuppressors, chemotherapy, steroids, and finasteride) during the previous 6 months, smoking and bacterial urogenital infection (evaluated by quantitative sperm and urethral swab culture), and previous chemotherapy.

At baseline, all patients were checked for LH, FSH, TT, albumin, DHEAS, 17*α*OH-P, SHBG, glycaemia, and insulin serum levels. The hormone measurement was performed by electrochemiluminescence (Hitachi-Roche equipment, Cobas 6000, Roche Diagnostics, Indianapolis, IN, USA). Free-T (fT) and bioavailable T (bio-T) were calculated using the Vermeulen formula [43], as recommended by a Task Force of the Endocrine Society [44]. IR was evaluated through the calculation of the HOMA index (fasting plasma glucose (mg/dl) × insulin (*μ*UI/l)/405), and a value above 2.5 was considered indicative of IR. Patients also underwent conventional (performed according to the WHO criteria [45]) and biofunctional sperm parameters, bioelectrical impedance analysis (BIA), and scrotal ultrasound scan. Left and right testicular volumes (TV) were also measured, and total TV was obtained through the summation of both TV. Monolateral TV was considered normal when it ranged between 15 and 25 ml [46]. The BMI was calculated applying the formula: weight (kg)/(height (m) × height (m)).

Among early AGA patients, two groups were identified: Group 1, having one of the following parameters BMI >25 kg/m^2^, IR, and/or SHBG <25 nmol/l or a combination thereof, and Group 2, negative for such findings.

The protocol was approved by the internal International Review Board, and an informed written consent was obtained from each patient. This work was carried out in accordance with the Declaration of Helsinki.

### 2.1. Statistical Analysis

The data were analyzed by Student's *t*-test and chi-squared test, as appropriate. A *P* value lower than 0.05 was accepted as statistically significant. A trend was assumed for *P* values ranging from 0.05 to 0.099.

## 3. Results

**Figure 1:**
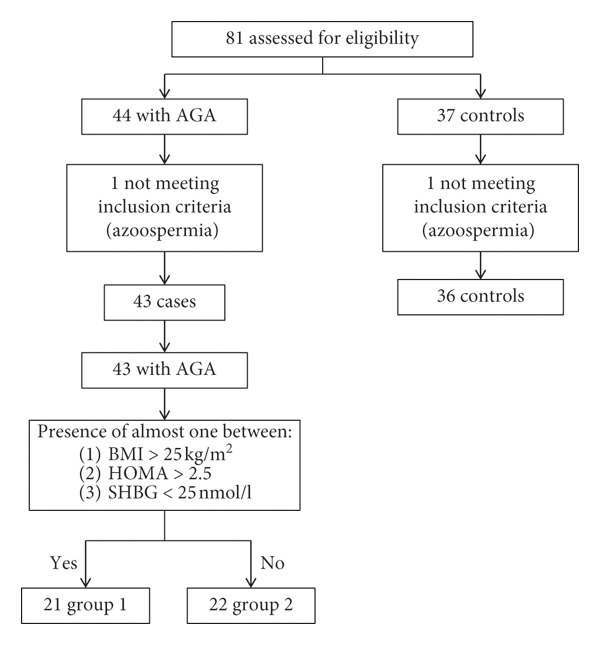
Study design. Eighty-one men younger than thirty years were enrolled in this study. Among them, forty-three had androgenetic alopecia (AGA) and were recruited as patients, and thirty-six made up the group of controls. Patients with AGA and at least one between BMI >25 kg/m^2^, HOMA index >2.5, and SHBG <25 nmol/l were included in Group 1 (*n* = 21). The remaining men made up Group 2 (*n* = 22). Group 1 included 7 patients having BMI >25 kg/m^2^, 2 patients having SHBG <25 nmol/l, 4 with IR, and 8 a combination of such alterations.

Eighty-one men were evaluated for eligibility, and two of them were excluded because they had azoospermia. Forty-three men with early-onset AGA (mean age: 24.3 ± 0.5 years; range: 14–30 years) were recruited and matched for age to thirty-six healthy subjects (mean age: 23.5 ± 0.5 years; range: 18–29 years) with no sign of AGA (Figure 1). The mean age of AGA onset was 20.8 ± 3.0 years (range: 16–26 years).

All patients with early AGA had significantly higher BMI and 17*α*OH-P serum levels compared to controls (*P* < 0.05); an upward trend for DHEAS, a downward trend for TT, and a higher sperm apoptosis percentage compared to controls (*P* < 0.05) were also found. The hormone/metabolic profile, conventional and biofunctional sperm parameters, and TV of patients and controls are summarized in Tables 2 and 3.

Among early AGA patients, we identified Group 1 (*n* = 21), having one of the following parameters BMI >25 kg/m^2^ (*n* = 7), IR (*n* = 4), and/or SHBG <25 nmol/l (*n* = 2) or a combination thereof (*n* = 8), and Group 2 (*n* = 22), negative for such parameters (Figure 1).

Results from Group 1 compared to Group 2 showed significantly higher levels of insulin (*P* < 0.01) and LH (*P* < 0.05); lower TT levels (*P* < 0.05) and smaller left TV (*P* < 0.05); an upward trend for the percentage of fat mass, triglycerides (TGL), and sperm progressive motility; and a downward trend for the percentage of fat-free mass, total TV, and sperm concentration.

Results from Group 1 compared to controls showed significantly higher percentage of fat mass (*P* < 0.05), DHEAS (*P* < 0.05), and seminal fluid (*P*=0.027); lower TT (*P*=0.016), left TV (*P* < 0.05), and leukocyte concentration (*P* < 0.05); an upward trend for LH, 17*α*OH-P levels; and the percentage of spermatozoa in apoptosis. Notably, Group 1 showed a lower but not significant different value of the right TV (14.6 ± 2.6 ml).

**Figure 2:**
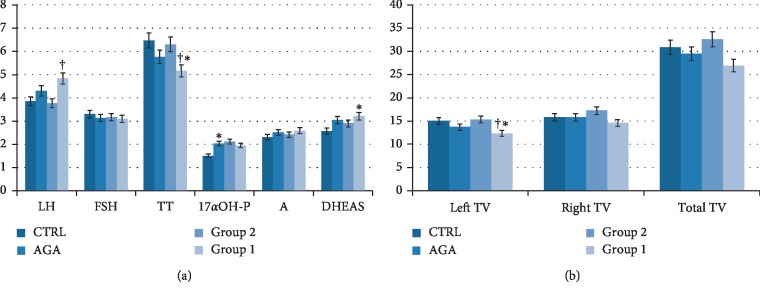
Hormonal parameters in Group 1, Group 2, AGA, and control group (a). Patients with early-onset androgenetic alopecia (AGA) had higher 17*α*-hydroxyprogesterone (17*α*OH-P) and an uptrend towards DHEAS values compared to controls. Group 1 had lower total testosterone (TT) values compared both to Group 2 and to controls. ^*∗*^*P* < 0.05 compared to control; ^†^*P* < 0.05 compared to Group 2 (Student's *t*-test). Left, right, and total testicular volume in Group 1, Group 2, AGA, and control group (b). No difference in total, left, and right testicular volume (TV) was observed in patients with early-onset AGA compared to controls. Group 1 showed lower left TV compared both to Group 2 and to controls. A downward trend for total TV was found in patients of Group 1 compared to those of Group 2. ^*∗*^*P* < 0.05 compared to control; ^†^*P* < 0.05 compared to Group 2 (Student's *t*-test).

As far the conventional sperm parameters, Group 1 showed an increased volume compared with Group 2 and controls (*P* < 0.05), a downward trend towards a lower sperm concentration compared to Group 2 (0.05 < *P* < 0.1), an upward trend towards an increased total motility compared to controls (0.05 < *P* < 0.1), and lower concentration of leukocytes in the seminal fluid compared to controls (*P* < 0.05) (Table 3). Finally, the biofunctional sperm parameters showed that the percentage of apoptotic spermatozoa was higher in patients with AGA compared to controls (*P* < 0.05) (Table 3). Results are summarized in Figure 2.

## 4. Discussion

Early-onset AGA has been proposed as a phenotypic sign of the male PCOS-equivalent. Previous studies have described the presence of a hormonal PCOS-like pattern in men with early-onset AGA, including low levels of SHBG [20–22, 24, 29, 47] and FSH [20–22, 29], and increased levels of 17*α*OH-P [21] and DHEAS [23, 24]. Data on TT and fT serum levels are contradictory: low fT levels [19, 28], increased free-androgen index (FAI) [20, 21, 29, 48], or subnormal TT [20, 21] values have been described. Patients may also show hyperglycaemia [40], IR [24–28], hyperinsulinemia [21, 47], metabolic syndrome [47], hypercholesterolemia [28], higher mean diastolic blood pressure [25, 26], and higher BMI levels [24]. An increased prevalence of T2DM and CVDs has also been reported in these men [5]. Since women with PCOS also present similar metabolic abnormalities and a higher risk for CVDs [49], these findings seem to support the hypothesis of the existence of a male PCOS-equivalent.

Male PCOS-equivalent may be considered as a “new” or previously unrecognized endocrine syndrome affecting the male gender. The delay in its recognition might be due to the sex-related perception of the phenotypic signs. Accordingly, suggested features include clinical signs of hyperandrogenism, such as early-onset AGA, acne, and/or hypertrichosis. While women readily perceive irregular menses, hirsutism, acne, and/or defluvium, early-onset AGA may be seen as a part of the normal masculine virilization [5]. Other features occurring before the age of 35 include PCOS-like hormonal pattern, metabolic abnormalities, and/or trend towards higher BMI values [5]. A family history positive for female PCOS may be also suggestive for the syndrome, which may expose to higher risk of developing DM II, CVD, and prostate diseases later in life [5].

In this context, the pathogenesis of early-onset AGA has been addressed to the peripheral conversion of weak adrenocortical androgens (e.g., DHEAS) into stronger androgens. Furthermore, hyperinsulinemia or IR could promote microcirculatory insufficiency, hypoxia, and hair follicle miniaturization into the scalp [5]. However, insulin could also negatively impact on testicular steroidogenesis, decreasing testosterone production [50], and on Sertoli cell function as well [51]. Hence, hyperinsulinemia might hypothetically lead to increase of DHEAS and decrease of TT serum levels.

Not all women with clinical signs of hyperandrogenism satisfy female PCOS diagnostic criteria. Similarly, it is likely that not all men with early-onset AGA are affected by the syndrome. Thus, additional markers are needed to identify those who may have the male PCOS-equivalent syndrome among men with early-onset AGA.

Based on previous reports that showed the presence of IR, higher BMI, and lower SHBG levels in men with early-onset AGA [19, 21, 24–28], we classified patients into two groups: those with at least one among these signs: BMI >25 Kg/m^2^, IR and/or SHBG <25 nmol/l (Group 1) and those who did not have any of them (Group 2). The TV values found in Group 1 support the hypothesis that patients with early-onset AGA who might have a male PCOS-equivalent syndrome do show a distinct gonadal profile compared with their not-at-risk counterparts.

As for the spermatogenetic testicular function, a downward trend for sperm concentration in Group 1 compared to Group 2 was found, but serum FSH levels did not differ significantly among all groups. However, abnormal levels of FSH have been described both in men with early-onset AGA and to controls [19–21, 46] and in first-degree male relatives of women with PCOS [52].

Group 1 also showed higher fat mass percentage compared to controls and an upward trend compared to Group 2, in agreement with a previous study [53].

These findings support the possible role of BMI, IR, and SHBG as candidate diagnostic criteria to identify the male PCOS-equivalent syndrome among men with early-onset AGA. Indeed, the lower TT and TV levels, along with the higher LH values in patients with early-onset AGA and at least one among BMI >25 Kg/m^2^, IR, and SHBG <25 nmol/l compared to controls indicate a worse gonadal profile in this subset of men. Similar to women [49], it might represent a feature of the male PCOS-equivalent. The occurrence of a worse gonadal function in these men may predispose to the development of CVDs [54, 55] and/or DM type II later in life, as it happens for women with PCOS [30–34]. In addition, a higher risk of CVDs has been described in men with AGA [56]. The early detection of hypogonadism may prevent the onset of such comorbidities.

## 5. Conclusions

The results of our study suggest the presence of a distinct gonadal profile in men with early-onset AGA and presenting with at least one among BMI >25 Kg/m^2^, IR, and SHBG <25 nmol/l. Similarly, to the female PCOS which negatively impact on the ovarian function, such distinct gonadal profile may be associated to a male PCOS-equivalent in a subset of patients, including increased DHEAS and reduced TT serum levels. Therefore, patients with early-onset AGA for increased BMI, IR, and/or SHBG serum levels should undergo to a more accurate control of the gonadal function, although larger and well-powered studies are needed to confirm our findings.

## Figures and Tables

**Table 1:** Clinical, biochemical and anamnestic features of the male equivalent of PCOS [5].

Age	Features of the male PCOS-equivalent
<35 years	(i) Clinical signs of hyperandrogenism (early-onset AGA and/or acne and/or hypertrichosis) (ii) PCOS-like hormonal pattern (increased DHEAS, AMH, 17*α*-OH-progesterone, FAI, decreased FSH) (iii) Metabolic abnormalities (insulin resistance, low SHBG levels, hyperglycaemia, hyperinsulinemia) and/or a trend towards higher BMI values (iv) A familiar history positive for PCOS
Elderly men	Diabetes mellitus, cardiovascular diseases, benign prostatic hyperplasia, prostatitis, prostate cancer

**Table 2:** Clinical features and serum hormone levels in patients with early-onset androgenetic alopecia (AGA) and in controls.

Parameter	Patients with AGA: *n* = 43	Group 1^a^: *n* = 21	Group 2^b^: *n* = 22	Control subjects: *n* = 36
Age (year)	24.3 (3.4)	24.0 (3.9)	24.5 (2.8)	23.3 (2.7)
BMI (kg/m^2^)	25.5 (3.8)^*∗*^	27.9 (3.8)^*∗*†^	23.0 (1.6)^*∗*^	23.7 (3)
Fat mass (%)	15.6 (4.8)	17.5 (4.1)^*∗*^^d^	12.4 (4.7)	13.2 (5.3)
Cholesterol (mg/dl)	167.5 (31.4)	163.6 (35.4)	171.2 (27.8)	170.1 (38.2)
HDL cholesterol (mg/dl)	49.2 (13.3)	45.4 (10.9)	52.7 (14.6)	47.7 (9.1)
LDL cholesterol (mg/dl)	104.4 (25.0)	101.7 (28.5)	106.9 (21.9)	100.4 (45.6)
Triglycerides (mg/dl)	69.9 (38.5)	82.5 (46.0)^b^	58.1 (26.1)	68.7 (35.6)
Glycaemia (mg/dl)	84.4 (8.0)	85.1 (8.9)	83.7 (7.1)	83.8 (6.1)
Insulin (*μ*U/ml)	8.8 (6.2)	11.8 (7.7)^†^	6.0 (2.1)^*∗*^	7.3 (3.2)
HOMA index	1.9 (1.4)	2.5 (1.8)^*∗*†^	1.3 (0.5)^*∗*^	1.5 (0.7)
LH (mIU/ml)	4.3 (1.8)	4.9 (2.1)^†^	3.8 (1.4)	3.9 (2.2)
FSH (mIU/ml)	3.1 (2.0)	3.1 (1.8)	3.2 (2.2)	3.3 (2.5)
TT (ng/ml)	5.8 (1.71)^b^	5.2 (1.7)^*∗*†^	6.3 (1.6)	6.5 (2.0)
17*β*-Estradiol (pg/ml)	24.5 (9.6)	22.9 (10.4)	26.0 (8.7)	25.6 (7.7)
Androstenedione (ng/ml)	2.5 (1.1)	2.6 (1.2)	2.4 (1.0)	2.3 (0.9)
DHEAS (*μ*g/dl)	304 (17.1)^d^	321.5 (112.6)	290.3 (97.0)	257.8 (107.1)
17*α*OH-P (ng/ml)	2.1 (0.9)^†*∗*^	2.0 (1.1)^c^	2.1 (0.7)	1.5 (0.6)
Cortisol (nmol/l)	429.6 (125.3)	429.8 (132.9)	429.3 (120.4)	383.1 (119.0)
SHBG (nmol/l)	32.9 (10.3)	28.9 (9.7)^d^	36.6 (9.9)	34.7 (13.7)
Bio-T (%)	44.8 (8.1)	46.9 (7.6)	43.0 (8.4)	45.3 (11.1)
Free-T	1.8 (0.4)	2.0 (0.3)	1.7 (0.4)	1.8 (0.5)

Abbreviations: 17*α*OH-P = 17*α*-hydroxyprogesterone; Bio-T = bioavailable testosterone; BMI = body mass index; Free-T = free testosterone; FSH = follicle-stimulating hormone; LH = luteinizing hormone; SHBG = Sex hormone-binding globulin; TT = total testosterone. ^a^Patients with early-onset AGA with at least one of the following parameters: body mass index (BMI) >25 kg/m^2^, insulin-resistance (IR), and/or sex hormone-binding globulin (SHBG) <25 nmol/l. ^b^Patients with early-onset AGA which did not satisfy any of the aforementioned criteria. ^*∗*^*P* < 0.05 compared to controls; ^†^*P* < 0.05 compared to Group 2; ^c^*P* < 0.1 compared to controls; ^d^*P* < 0.1 compared to Group 2 (Student's *t*-test). Results are expressed as mean value (standard deviation).

**Table 3:** Sperm parameters and testicular volume in patients with early-onset androgenetic alopecia (AGA) and in controls.

Parameters	Patients with AGA: *n* = 43	Group 1^a^: *n* = 21	Group 2^b^: *n* = 22	Control subjects: *n* = 36
*Conventional sperm parameters*				
Volume (ml)	3.6 (2.2)	4.2 (2.8)^*∗*†^	3.0 (1.5)	2.8 (1.3)
pH	7.9 (1.3)	8.0 (0.3)	7.7 (1.8)	8.0 (0.3)
Sperm concentration (mil/ml)	66.6 (49.8)	51.7 (42.4)^d^	79.9 (53.1)	52.4 (33.2)
Total sperm count (mil/ejaculate)	193.8 (142.8)	51.7 (42.7)	79.9 (53.1)	147.7 (104.3)
Forward motility (%)	18.7 (10.6)	21.3 (10.2)	16.3 (10.6)	15.9 (10.8)
Total motility (%)	56.6 (14.5)	60.9 (9.9)^c^	52.8 (17.0)	59.5 (8.3)
Normal morphology (%)	6.3 (2.9)	7.2 (3.0)	5.6 (2.7)	5.8 (3.3)
Leukocyte concentration (mil/ml)	2.1 (3.8)	1.2 (1.2)	3.0 (5.0)	2.5 (5.7)
Total leukocyte count (mil/ejaculate)	2.9 (8.2)	0.6 (0.7)^*∗*^	5.1 (11.1)	2.7 (3.6)

*Biofunctional sperm parameters*				
Low mitochondrial membrane potential (%)	29.0 (20.8)	26.2 (17.5)	31.4 (23.6)	19.8 (17.8)
Abnormal chromatin compactness (%)	25.1 (8.1)	25.7 (5.7)	24.8 (9.5)	25.9 (8.2)
Alive spermatozoa (%)	73 (12.0)	72.2 (12.9)	73.8 (11.6)	75.5 (14.2)
Phosphatidylserine externalization (%)	1.9 (1.9)	2.4 (2.3)	1.5 (1.3)	1.8 (1.5)
Sperm apoptosis (%)	10.7 (12.6)^*∗∗*^	7.8 (7.5)^c,d^	13.5 (16.0)	3.4 (3.7)
DNA fragmentation (%)	2.9 (3.5)	1.7 (1.1)	4.2 (4.8)	1.2

*Testicular volume*				
Total testicular volume (ml)	29.5 (7.5)	24.9 (4.7)	32.6 (9.1)	30.9 (7.9)
Left testicular volume (ml)	13.7 (3.7)	12.3 (2.8)^*∗*†^	15.4 (4.1)	15.0 (4.3)
Right testicular volume (ml)	15.8 (4.3)	14.6 (2.6)	17.2 (5.6)	15.8 (4.0)

^a^Patients with early-onset AGA with at least one of the following parameters: body mass index (BMI) >25 Kg/m^2^, insulin-resistance (IR), and/or sex hormone-binding globulin (SHBG) <25 nmol/l. ^b^Patients with early-onset AGA which did not satisfy any of the aforementioned criteria. ^*∗*^*P* < 0.05 compared to controls; ^†^*P* < 0.05 compared to Group 2; ^c^*P* < 0.1 compared to controls; ^d^*P* < 0.1 compared to Group 2 (Student's *t*-test). The DNA fragmentation was evaluated through TUNEL test. Results are expressed as mean value (standard deviation).

## Data Availability

Data used to support the findings of this study have not been made available because they belong to a wider project on the male PCOS-equivalent that is currently going on.

## Abbreviations

AGA: Androgenetic alopecia;
bio-T: Bio-available T;
BIA: Bioelectrical impedance analysis;
BMI: Body mass index;
CVDs: Cardiovascular diseases;
DHEAS: Dehydroepiandrosterone sulfate;
DM II: Type II diabetes mellitus;
FAI: Free androgen index;
FSH: Follicle-stimulating hormone;
fT: Free testosterone;
17*α*OH-P: 17*α*-hydroxyprogesterone;
IR: Insulin resistance;
LH: Luteinizing hormone;
PCOS: Polycystic ovary syndrome;
SHBG: Sex hormone-binding globulin;
TGL: Triglycerides;
TT: Total testosterone;
TV: Testicular volume.

## References

[1] Isidori A. M., Balercia G., Calogero A. E. (2015). Outcomes of androgen replacement therapy in adult male hypogonadism: recommendations from the Italian society of endocrinology. *Journal of Endocrinological Investigation*.

[2] Hamilton J. B. (1951). Patterned loss of hair in man: types and incidence. *Annals of the New York Academy of Sciences*.

[3] Norwood O. T. (1975). Male pattern baldness: classification and incidence. *Southern Medical Journal*.

[4] Cannarella R., La Vignera S., Condorelli R. A., Calogero A. E. (2017). Glycolipid and hormonal profiles in young men with early-onset androgenetic alopecia: a meta-analysis. *Scientific Reports*.

[5] Cannarella R., Condorelli R. A., Mongioì L. M., La Vignera S., Calogero A. E. (2018). Does a male polycystic ovarian syndrome equivalent exist?. *Journal of Endocrinological Investigation*.

[6] Legro R. S., Driscoll D., Strauss J. F., Fox J., Dunaif A. (1998). Evidence for a genetic basis for hyperandrogenemia in polycystic ovary syndrome. *Proceedings of the National Academy of Sciences*.

[7] Legro R. S., Finegood D., Dunaif A. (1998). A fasting glucose to insulin ratio is a useful measure of insulin sensitivity in women with polycystic ovary syndrome 1. *The Journal of Clinical Endocrinology & Metabolism*.

[8] Legro R. S., Kunselman A. R., Demers L., Wang S. C., Bentley-Lewis R., Dunaif A. (2002). Elevated dehydroepiandrosterone sulfate levels as the reproductive phenotype in the brothers of women with polycystic ovary syndrome. *The Journal of Clinical Endocrinology & Metabolism*.

[9] Franks S., McCarthy M. (2004). Genetics of ovarian disorders: polycystic ovary syndrome. *Reviews in Endocrine and Metabolic Disorders*.

[10] Calogero A. E., Calabrò V., Catanuso M., Condorelli R. A., La Vignera S. (2011). Understanding polycystic ovarian syndrome pathogenesis: an updated of its genetic aspects. *Journal of Endocrinological Investigation*.

[11] Lunde O., Magnus P., Sandvik L., Høglo S. (1989). Familial clustering in the polycystic ovarian syndrome. *Gynecologic and Obstetric Investigation*.

[12] Benítez R., Sir-Petermann T., Palomino A. (2001). Prevalence of metabolic disorders among family members of patients with polycystic ovary syndrome. *Revista Medica de Chile*.

[13] Yilmaz M., Bukan N., Ersoy R. (2005). Glucose intolerance, insulin resistance and cardiovascular risk factors in first degree relatives of women with polycystic ovary syndrome. *Human Reproduction (Oxford, England)*.

[14] Norman R. J., Masters S., Hague W. (1996). Hyperinsulinemia is common in family members of women with polycystic ovary syndrome. *Fertility and Sterility*.

[15] Kaushal R., Parchure N., Bano G., Kaski J.-C., Nussey S. S. (2004). Insulin resistance and endothelial dysfunction in the brothers of Indian Subcontinent Asian women with polycystic ovaries. *Clinical Endocrinology*.

[16] Carey A. H., Chan K. L., Short F., White D., Williamson R., Franks S. (1993). Evidence for a single gene effect causing polycystic ovaries and male pattern baldness. *Clinical Endocrinology*.

[17] Govind A., Obhrai M. S., Clayton R. N. (1999). Polycystic ovaries are inherited as an autosomal dominant trait: analysis of 29 polycystic ovary syndrome and 10 control families. *The Journal of Clinical Endocrinology & Metabolism*.

[18] Legro R. S. (2000). Is there a male phenotype in polycystic ovary syndrome families?. *Journal of Pediatric Endocrinology and Metabolism*.

[19] Starka L., Duskova M., Cermakova I., Vrbiková J., Hill M. (2005). Premature androgenic alopecia and insulin resistance. Male equivalent of polycystic ovary syndrome?. *Endocrine Regulations*.

[20] Stárka L., Čermáková I., Dušková M., Hill M., Doležal M., Poláček V. (2004). Hormonal profile of men with premature balding. *Experimental and Clinical Endocrinology & Diabetes*.

[21] Dusková M., Cermáková I., Hill M., Vanková M., Sámalíková P., Stárka L. (2004). What may be the markers of the male equivalent of polycystic ovary syndrome?. *Physiological research*.

[22] Pitts R. L. (1987). Serum elevation of dehydroepiandrosterone sulfate associated with male pattern baldness in young men. *Journal of the American Academy of Dermatology*.

[23] Sanke S., Chander R., Jain A., Garg T., Yadav P. (2016). A comparison of the hormonal profile of early androgenetic alopecia in men with the phenotypic equivalent of polycystic ovarian syndrome in women. *JAMA Dermatology*.

[24] Matilainen V., Koskela P., Keinänen-Kiukaanniemi S. (2000). Early androgenetic alopecia as a marker of insulin resistance. *The Lancet*.

[25] González-González J. G., Mancillas-Adame L. G., Fernández-Reyes M. (2009). Androgenetic alopecia and insulin resistance in young men. *Clinical Endocrinology*.

[26] Arias-Santiago S., Gutiérrez-Salmerón M. T., Buendía-Eisman A., Girón-Prieto M. S., Naranjo-Sintes R. (2011). Sex hormone-binding globulin and risk of hyperglycemia in patients with androgenetic alopecia. *Journal of the American Academy of Dermatology*.

[27] Su L.-H., Chen T. H.-H. (2010). Association of androgenetic alopecia with metabolic syndrome in men: a community-based survey. *British Journal of Dermatology*.

[28] Mumcuoglu C., Ekmekci T. R., Ucak S. (2011). The investigation of insulin resistance and metabolic syndrome in male patients with early-onset androgenetic alopecia. *European Journal of Dermatology*.

[29] Narad S., Pande S., Chari S., Gupta M. (2013). Hormonal profile in Indian men with premature androgenetic alopecia. *International Journal of Trichology*.

[30] Ehrman D. A., Barnes R. B., Rosenfield R. L., Cavaghan M. K., Imperial J. (1999). Prevalence of impaired glucose tolerance and diabetes in women polycystic ovary syndrome. *Diabetes Care*.

[31] Legro R. S., Kunselman A. R., Dodson W. C., Dunaif A. (1999). Prevalence and predictors of risk for type 2 diabetes mellitus and impaired glucose tolerance in polycystic ovary syndrome: a prospective, controlled study in 254 affected women 1. *Journal of Clinical Endocrinology & Metabolism*.

[32] Wild R. A., Painter R. D., Coulson P. B., Carruth K. B., Ranney R. B. (1985). Lipoprotein lipid concentrations as cardiovascular risk in patients with polycystic ovary syndrome. *The Journal of Clinical Endocrinology and Metabolism*.

[33] Wild S., Pierpoint T., McKeigue H., Jacobs H. (2000). Cardiovascular disease in women with polycystic ovary syndrome at long-term follow-up: a retrospective cohort study. *Clinical Endocrinology*.

[34] Legro R. S. (2003). Polycystic ovary syndrome and cardiovascular disease: a premature association?. *Endocrine Reviews*.

[35] Malipatil N. S., Yadegarfar G., Lunt M. (2019). Male hypogonadism: 14-year prospective outcome in 550 men with type 2 diabetes. *Endocrinology, Diabetes & Metabolism*.

[36] Saad F., Caliber M., Doros G., Haider K. S., Haider A. (2020). Long-term treatment with testosterone undecanoate injections in men with hypogonadism alleviates erectile dysfunction and reduces risk of major adverse cardiovascular events, prostate cancer, and mortality. *The Aging Male*.

[37] Sinclair R. D., Dawber R. P. R. (2001). Androgenetic alopecia in men and women. *Clinics in Dermatology*.

[38] Knochenhauer E. S., Key T. J., Kahsar-Miller M., Waggoner W., Boots L. R., Azziz R. (1998). Prevalence of the polycystic ovary syndrome in unselected black and white women of the southeastern United States: a prospective study. *Journal of Clinical Endocrinology & Metabolism*.

[39] Diamanti-Kandarakis E., Kouli C. R., Bergiele A. T. (1999). A survey of the polycystic ovary syndrome in the Greek Island of Lesbos: hormonal and metabolic profile. *The Journal of Clinical Endocrinology & Metabolism*.

[40] Asuncion M., Calvo R. M., San Millán J. L., Sancho J., Avila S., Escobar-Morreale H. F. (2000). A prospective study of the prevalence of the polycystic ovary syndrome in unselected Caucasian women from Spain. *Journal of Clinical Endocrinology & Metabolism*.

[41] Azziz R., Woods K. S., Reyna R., Key T. J., Knochenhauer E. S., Yildiz B. O. (2004). The prevalence and features of the polycystic ovary syndrome in an unselected population. *The Journal of Clinical Endocrinology & Metabolism*.

[42] Lacarrubba F., Micali G., Tosti A. (2015). Scalp dermoscopy or trichoscopy. *Alopecias—Practical Evaluation and Management*.

[43] Vermeulen A., Verdonck L., Kaufman J. M. (1999). A critical evaluation of simple methods for the estimation of free testosterone in serum. *The Journal of Clinical Endocrinology & Metabolism*.

[44] Bhasin S., Cunningham G. R., Hayes F. J. (2010). Testosterone therapy in men with androgen deficiency syndromes: an endocrine society clinical practice guideline. *The Journal of Clinical Endocrinology & Metabolism*.

[45] WHO (2010). *Laboratory Manual for the Examination and Processing of Human Semen*.

[46] Foresta C., Garolla A., Frigo A. C. (2013). Anthropometric, penile and testis measures in post-pubertal Italian males. *Journal of Endocrinological Investigation*.

[47] Pengsalae N., Tanglertsampan C., Phichawong T., Lee S. (2013). Association of early-onset androgenetic alopecia and metabolic syndrome in Thai men: a case-control study. *Journal of the Medical Association of Thailand*.

[48] Dusková M., Hill M., Stráka L. (2007). The polycystic ovary syndrome and its male equivalent. *CasLek Cesk*.

[49] Goodman N. F., Cobin R. H., Futterweit W., Glueck J. S., Legro R. S., Carmina E. (2015). American Association of Clinical Endocrinologists, American College of Endocrinology, and Androgen Excess and PCOS Society Disease State Clinical review: guide to the best practices in the evaluation and treatment of polycystic ovary syndrome—part 1. *Endocrine Practice*.

[50] Ahn S. W., Gang G.-T., Kim Y. D. (2013). Insulin directly regulates steroidogenesis via induction of the orphan nuclear receptor DAX-1 in testicular Leydig cells. *Journal of Biological Chemistry*.

[51] Cannarella R., Arato I., Condorelli R. A. (2019). Effects of insulin on porcine neonatal Sertoli cell responsiveness to FSH *in vitro*. *Journal of Clinical Medicine*.

[52] Torchen L. C., Kumar A., Kalra B. (2016). Increased antimüllerian hormone levels and other reproductive endocrine changes in adult male relatives of women with polycystic ovary syndrome. *Fertility and Sterility*.

[53] Dumesic D. A., Akopians A. L., Madrigal V. K. (2016). Hyperandrogenism accompanies increased intra-abdominal fat storage in normal weight polycystic ovary syndrome women. *The Journal of Clinical Endocrinology & Metabolism*.

[54] Rosano G. M. C., Sheiban I., Massaro R. (2007). Low testosterone levels are associated with coronary artery disease in male patients with angina. *International Journal of Impotence Research*.

[55] Antonio L., Wu F. C. W., O’Neill T. W. (2015). Associations between sex steroids and the development of metabolic syndrome: a longitudinal study in European men. *The Journal of Clinical Endocrinology & Metabolism*.

[56] Park S.-Y., Oh S. S., Lee W.-S. (2016). Relationship between androgenetic alopecia and cardiovascular risk factors according to BASP classification in Koreans. *The Journal of Dermatology*.

